# Rapidly Progressive Painless Acute Aortic Dissection: A Case Report

**DOI:** 10.5070/M5.52348

**Published:** 2026-07-31

**Authors:** Justin Yang, Tyler Rigdon, Kristin Lewis

**Affiliations:** * University of California, Irvine, Department of Emergency Medicine, Orange, CA

## Abstract

Acute aortic dissection (AAD) typically presents with sudden, severe chest or back pain. This case highlights an atypical painless presentation that was rapidly progressive and lethal. A 55-year-old woman presented with generalized weakness, agitation, and shortness of breath, but without pain. Initial point-of-care ultrasound (POCUS) was reassuring, but persistent agitation and emerging abdominal tenderness prompted reexamination and repeat POCUS, which revealed evidence of aortic dissection. Computed tomography angiogram (CTA) confirmed the diagnosis of a Stanford Type A dissection. Despite aggressive resuscitative efforts, the patient’s condition deteriorated rapidly. This case emphasizes the importance of observant reassessment and repeating POCUS in atypical presentations of AAD because subtle clinical changes can reveal life-threatening conditions such as aortic dissection.

**Topics:**

Acute aortic dissection, abdominal aortic dissection, painless aortic dissection, atypical presentation, point-of-care ultrasound reassessment, diagnostic ultrasound.

**Figure:**
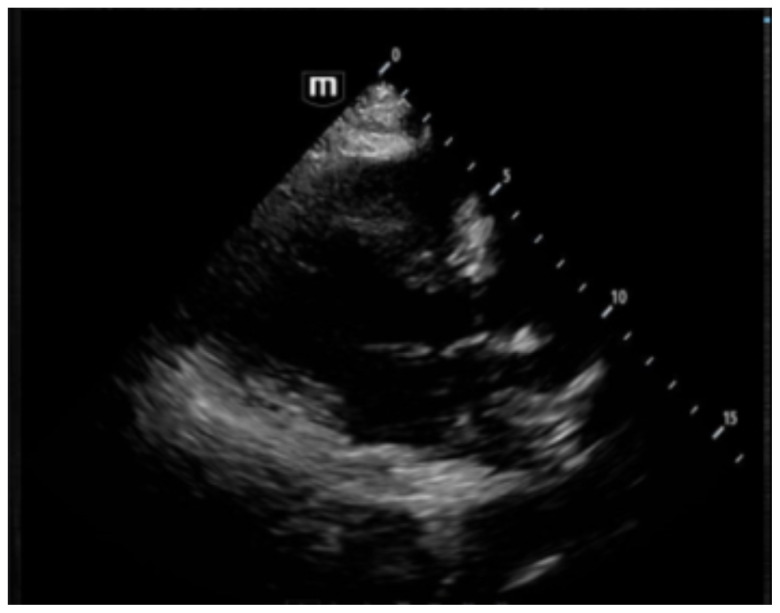


**Figure:**
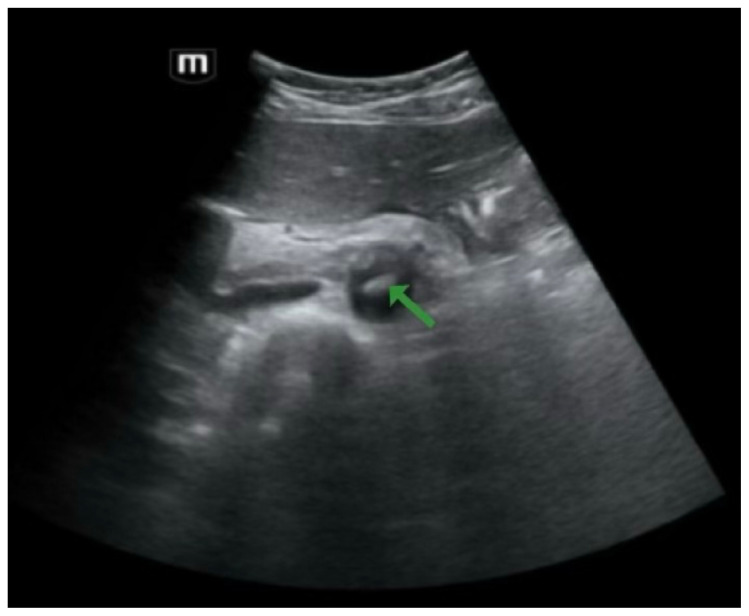


**Figure:**
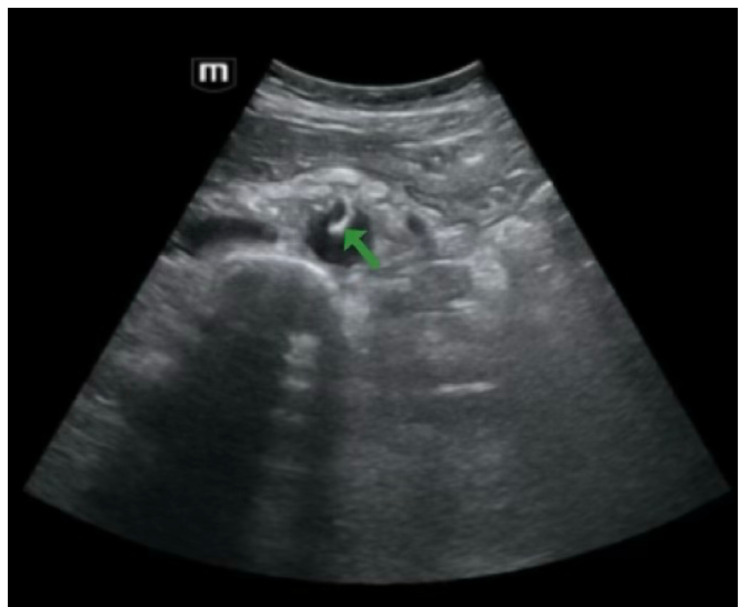


**Figure:**
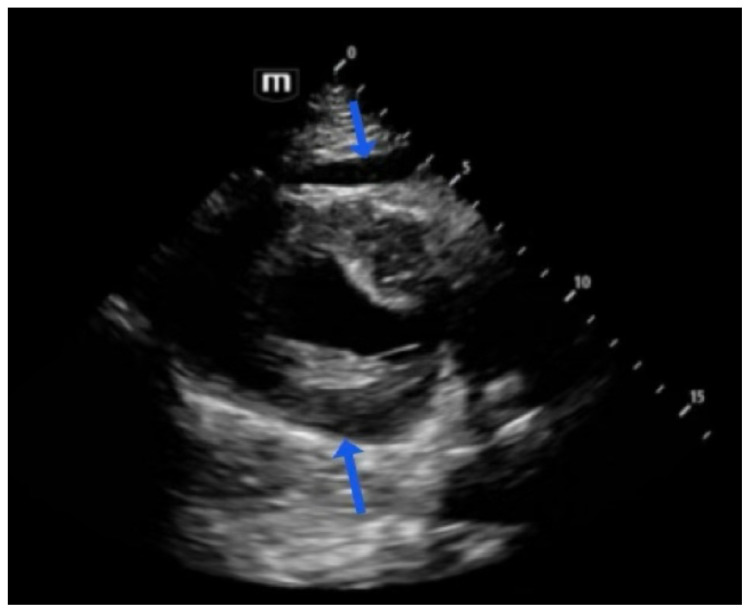


**Figure:**
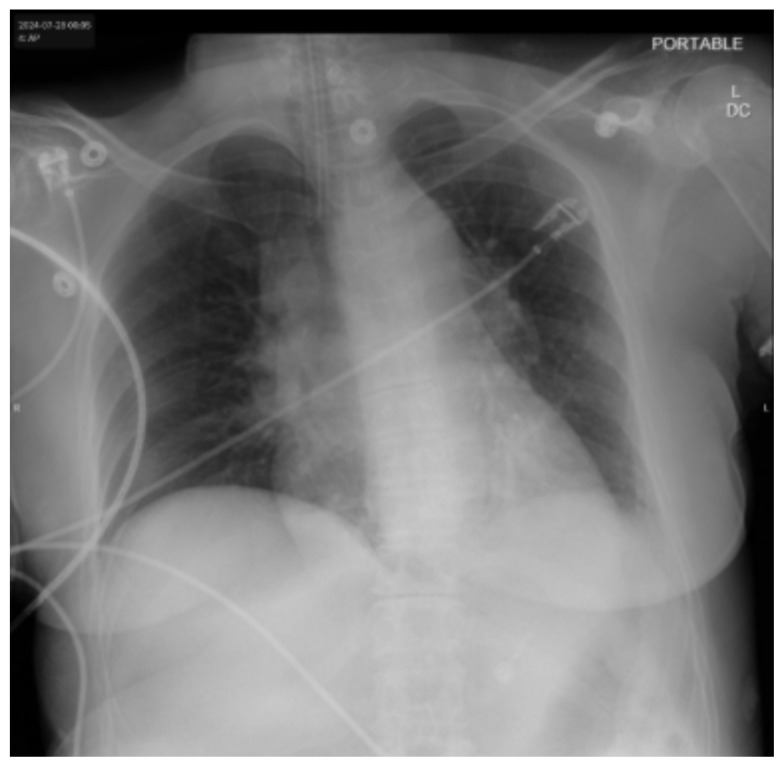


**Figure:**
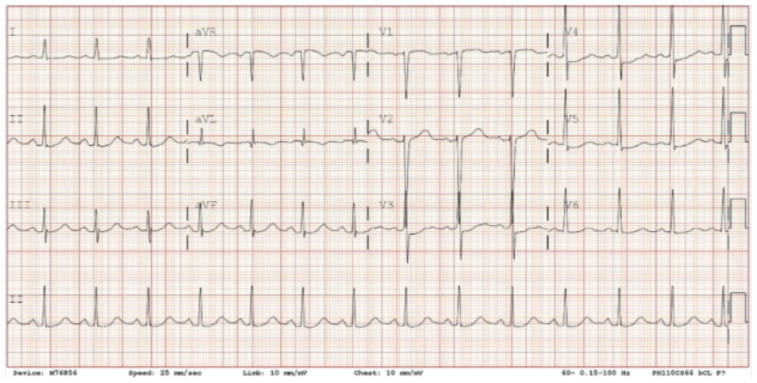


**Figure:**
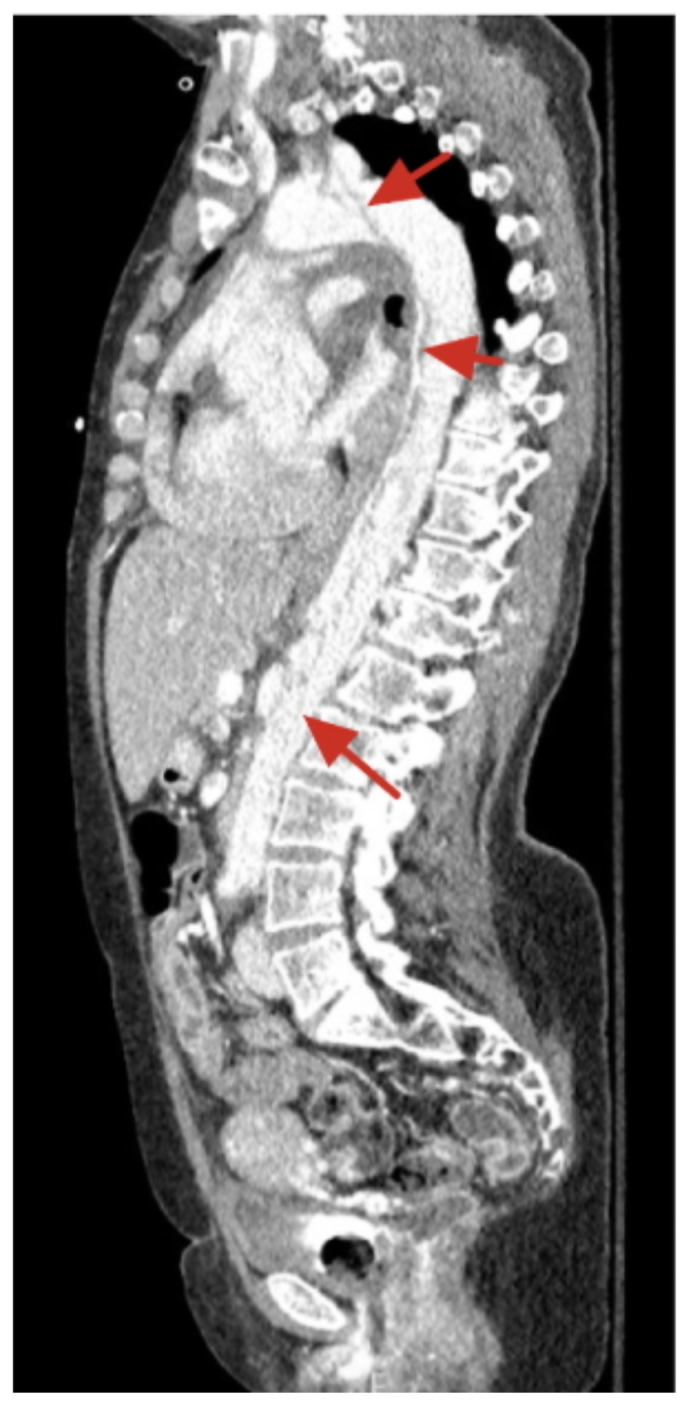


**Figure:**
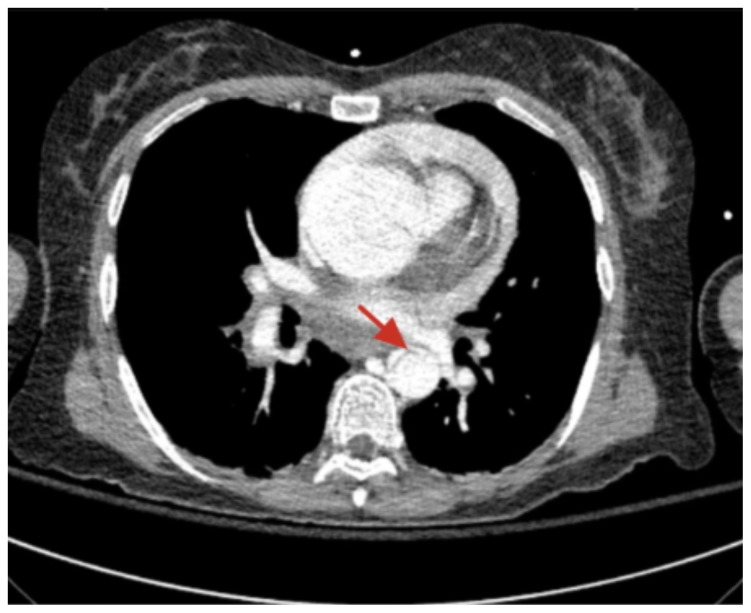


**Figure:**
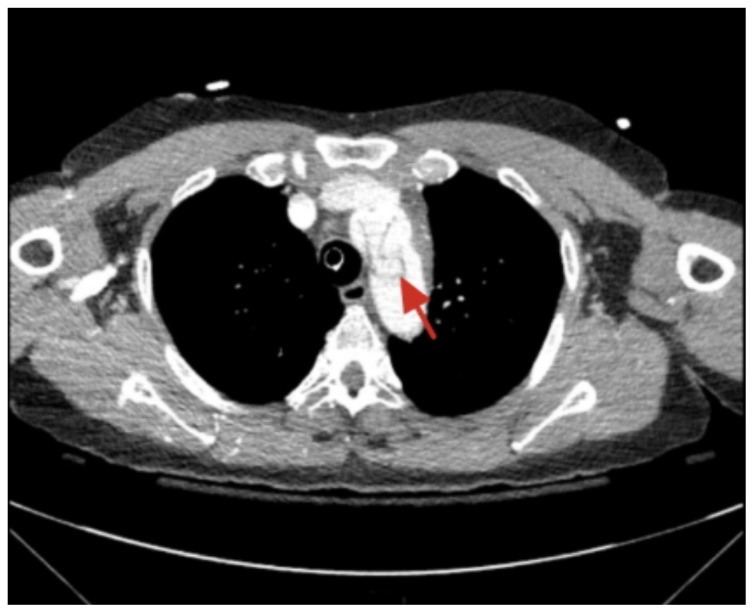


**Figure:**
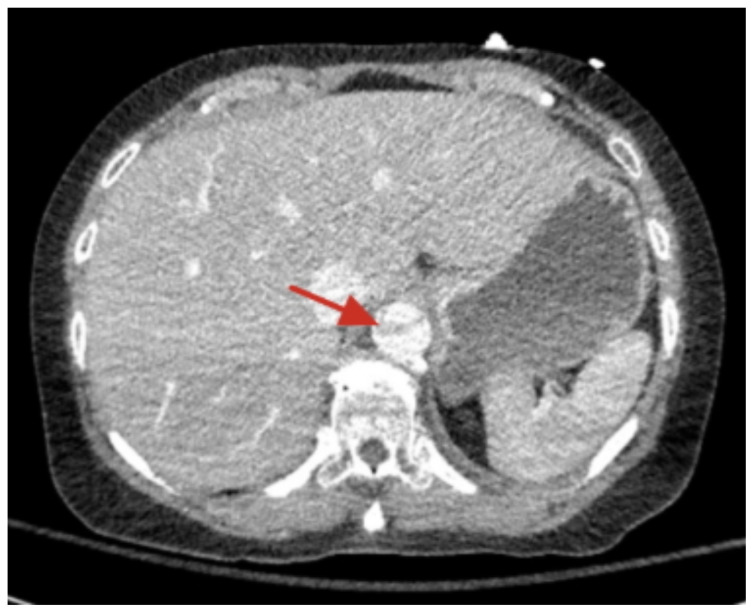


**Figure:**
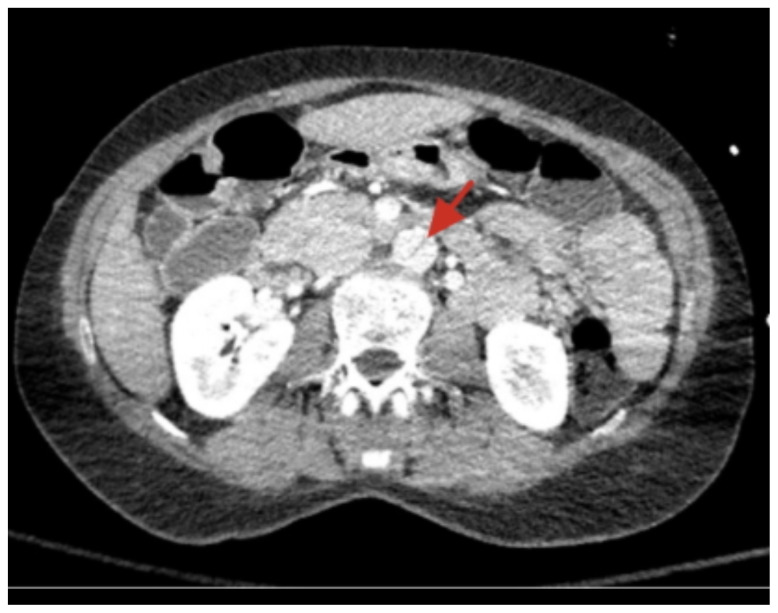


## Brief introduction

Acute aortic dissection is a rare but life-threatening condition characterized by a tear in the aortic intima, leading to separation of the vessel wall layers. While classic presentations involve sudden, severe chest or back pain, up to 6.4% of AAD can manifest without pain.^1^ Furthermore, a significant subset of patients may exhibit atypical symptoms, including neurological manifestations like agitation and altered mental status (AMS), with or without the classic pain symptoms. Up to 18.6% of patients with type A AAD initially present with neurological symptoms at onset.^2^ Such atypical presentations have been found to complicate timely diagnosis, ultimately delaying management.^3^ Therefore, continuous reassessment along with POCUS is vital, as initial presentations may evolve, and early recognition can significantly impact patient outcomes.

## Presenting concerns and clinical findings

A 55-year-old woman was brought to the emergency department (ED) by emergency medical services (EMS) for generalized weakness and shortness of breath. No known medical history, including hypertension, connective tissue disease, or prior history of stimulant or substance use, was reported. She was reported to be found outside of a motel, cool and diaphoretic, with a prehospital point-of-care (POC) glucose of 30 mg/dL and was hypotensive. Baseline mental status and last known well were unknown. She received 10% Dextrose and approximately 300 cc of intravenous fluids in the field, with improvement in glucose to the 120’s and stabilization of vital signs.

Upon arrival at the emergency department, she was alert and oriented but appeared restless and anxious. She repeatedly stated, “I feel like I can’t breathe,” though her oxygen saturation remained 97–100% on room air. She denied any chest pain, abdominal pain, back pain, headache, vision changes, trauma, or recent illness. She reported no alcohol, tobacco, or drug use, and had no medication history.

Initial vital signs were notable for a forehead temperature of 95.7°F, heart rate of 66 bpm, blood pressure 156/70 mmHg, and respiratory rate of 24. Physical exam revealed a patient in moderate distress, with cool, clammy extremities and a capillary refill time of about 2 seconds. Peripheral pulses were symmetric and palpable. Cardiac and lung exams were unremarkable. The abdomen was initially soft and nontender. Neurologically, the patient was oriented and able to follow commands, but minimally communicative beyond repeated complaints of dyspnea. She was spontaneously moving all four of her extremities.

## Significant findings

A bedside cardiac and pulmonary POCUS was performed early in the ED course and showed no pericardial effusion, no right heart strain, and normal lung sliding bilaterally. Due to agitation, the patient would not cooperate and refused an ECG and chest X-ray early in her ED course. Later, a repeat focused physical exam was performed, during which the patient had new abdominal tenderness. A POCUS of the abdomen at that time demonstrated findings concerning for a dissection flap in the abdominal aorta (green arrows), and a new mild to moderate pericardial effusion was seen on a repeat cardiac POCUS suggesting an evolving aortic dissection (blue arrows).

An ECG was obtained shortly after her repeat POCUS and showed subtle diffuse ST depressions without ST elevation myocardial infarction or other acute ischemic changes. After the patient was intubated, her chest x-ray suggested possible subtle loss of the aortic knob contour and mediastinal widening.

The patient’s CTA revealed a type A AAD with extension from the aortic root to the bilateral external and internal iliac vessels as well as the coronary arteries (red arrows).

## Patient course

After the initial physical exam was performed, a cardiac and pulmonary POCUS was done which showed no pericardial effusion, right heart strain, or pneumothorax. Blankets were applied and intravenous (IV) fluids were started. The patient continued to appear restless despite lorazepam 1mg x1 and IV olanzapine 2.5mg x2, but notably she was oriented and answering questions appropriately. She refused an EKG and chest x-ray and attempted to remove her IV. A venous blood gas resulted 10 minutes after her arrival with a pH of 7.44, CO^2^ 35, HCO3 23.6, and a base excess of 0.0. Labs later revealed mild high sensitivity troponin elevation (12), lactic acid of 3, but normal hemoglobin, white blood cell count, electrolytes, and kidney function. A urine drug screen resulted an hour later and was positive for amphetamines only.

A repeat exam after approximately two hours revealed abdominal tenderness, prompting a second bedside ultrasound, which showed a dissection flap in the abdominal aorta and a new small pericardial effusion, raising concern for an acute, rapidly evolving aortic dissection. Left arm BP was 104/51 with a documented 20–30 mmHg higher systolic pressure compared to the right arm. However, right arm values were not separately recorded.

The patient was intubated for airway protection and to facilitate emergent CT imaging in the setting of worsening agitation and was subsequently taken for emergent CTA. During CT, the patient gradually became hypotensive despite fluids, blood products, and pressors. Imaging confirmed the aortic dissection, showing signs of rapid progression and proximal involvement of the aortic arch extending into the right and left coronary arteries. Distally, there was extension into the descending thoracic aorta, abdominal aorta, bilateral common iliac, bilateral external iliac and left internal iliac arteries. Shortly after completing the CTA, she became bradycardic and pulseless, and cardiopulmonary resuscitation (CPR)/advanced cardiovascular life support (ACLS) was initiated. Despite temporary return of spontaneous circulation (ROSC), she lost pulses again and ultimately expired. Pericardiocentesis was considered but not performed due to futility. At the time, coordination with the surgery team and review of the CT imaging confirmed that the dissection was severe, and further resuscitation was deemed futile.

## Discussion

Acute aortic dissection occurs when an intimal tear allows blood in the aorta to penetrate the medial layer, creating a false lumen that can propagate along the vessel. The ascending aorta is the most frequent site of origin, and dissections here carry the highest mortality due to complications such as pericardial tamponade and aortic rupture.^4^ While classic teaching emphasizes sudden, tearing chest pain, up to 6.4% of patients can present without pain^1^. One study noted that 17% of their studied population initially had a painless presentation, and those painless initial presentations were significantly more likely to present with neurological signs (44% of the painless vs 6% in the painful group).^9^ Neurological symptoms were also found to be present in 18.6% of AAD cases in another study, and there were significantly fewer complaints of pain in the group demonstrating neurological symptoms^2^. As in our case, the initial absence of discrete pain and the focus of agitation and evidence of altered mental status contributed to the ambiguity of the initial presentation obscuring the diagnosis.

In this case, initial POCUS was unrevealing, which unfortunately can be the situation in up to 14% of patients with a final diagnosis of Type A AAD.^8^ When this patient began to deteriorate, repeat bedside ultrasound did show evidence of a dissection flap in the abdominal aorta and a new pericardial effusion which then shifted the diagnostic focus and expedited confirmatory imaging and consulting surgery. These sonographic signs are highly specific for dissection, with studies reporting a sensitivity of 93.2% and a specificity of 90.9% for the diagnosis of aortic dissection with POCUS if a pericardial effusion, intimal flap, or an aortic outflow tract diameter >35 mm is present.^5^

Computed tomography angiogram confirmed a Stanford Type A AAD extending from the iliac arteries to the aortic root and bilateral coronary arteries. Computed tomography angiogram remains the gold standard for diagnosis of AAD with a specificity of 98%.^6^ Ultrasound was the only tool available at the bedside that enabled rapid, high-accuracy detection of evolving pathology, which directly led to emergent interventions. Initial management of aortic dissection typically includes IV beta blockers to reduce aortic wall stress and analgesia to blunt sympathetic tone, with a goal systolic blood pressure of 90–110 mmHg and a heart rate of 60–80 bpm.^7^ However, this patient became hypotensive shortly after diagnosis, requiring blood transfusion, vasopressors, and emergent surgical evaluation. Unfortunately, the dissection had progressed rapidly, resulting in hemodynamic collapse before definitive intervention could occur.

This case illustrates the challenges of diagnosing aortic dissection, especially in patients who fall outside typical symptom profiles. Atypical presentations of AAD can result in misdiagnosis in 16–39% of cases. With mortality increasing 1–2% every hour after symptom onset, timely diagnosis is crucial.^5^ When the diagnosis is uncertain or the patient’s condition is not responding to treatment as expected, such as worsening agitation despite sedation and persistent symptoms despite correction of prehospital hypoglycemia, serial reassessment is critical. Urine toxicology positive for amphetamines further complicated the clinical picture, raising concern for sympathomimetic contribution to agitation and physiologic stress. In this case, this included consideration of repeat bedside POCUS, a readily available and high-yield diagnostic tool. Even when initial tests are inconclusive, vigilance and timely reassessment can reveal critical diagnoses that alter management and may improve outcomes.

## Supplementary Information






































